# Gene Classification Based on Amino Acid Motifs and Residues: The *DLX* (*distal-less*) Test Case

**DOI:** 10.1371/journal.pone.0005748

**Published:** 2009-06-01

**Authors:** Nuno A. Fonseca, Cristina P. Vieira, Jorge Vieira

**Affiliations:** 1 Instituto de Biologia Molecular e Celular (IBMC); University of Porto, Porto, Portugal; 2 CRACS-INESC Porto, Universidade do Porto, Porto, Portugal; American Museum of Natural History, United States of America

## Abstract

**Background:**

Comparative studies using hundreds of sequences can give a detailed picture of the evolution of a given gene family. Nevertheless, retrieving only the sequences of interest from public databases can be difficult, in particular, when working with highly divergent sequences. The difficulty increases substantially when one wants to include in the study sequences from many (or less well studied) species whose genomes are non-annotated or incompletely annotated.

**Methodology/Principal Findings:**

In this work we evaluate the usefulness of different approaches of gene retrieval and classification, using the *distal-less* (*DLX*) gene family as a test case. Furthermore, we evaluate whether the use of a large number of gene sequences from a wide range of animal species, the use of multiple alternative alignments, and the use of amino acids aligned with high confidence only, is enough to recover the accepted *DLX* evolutionary history.

**Conclusions/Significance:**

The canonical *DLX* homeobox gene sequence here derived, together with the characteristic amino acid variants here identified in the *DLX* homeodomain region, can be used to retrieve and classify *DLX* genes in a simple and efficient way. A program is made available that allows the easy retrieval of synteny information that can be used to classify gene sequences. Maximum likelihood trees using hundreds of sequences can be used for gene identification. Nevertheless, for the *DLX* case, the proposed *DLX* evolutionary is not recovered even when multiple alignment algorithms are used.

## Introduction

When performing comparative studies, the first step is often to collect the sequences of interest from very divergent species from public databases using BLAST. Nevertheless, when genes belong to large gene families, thus showing homology with many different genes, retrieving only the sequences of interest can be difficult. For instance, two rounds of genome duplication in the lineage leading to the common ancestor of jawed vertebrates, as well as an extra genome duplication event in the fish lineage [1] further complicates the sequence retrieval process.

In order to confirm the identity of the retrieved sequences, a phylogenetic approach should always be used. However, establishing the relationship of very divergent sequences can be a challenging task. For instance, different multiple sequence alignment (MSA) algorithms can produce different alignments, which in turn can influence the inferred phylogenetic reconstruction and thus lead to different conclusions. Furthermore, Essoussi *et al.* ([2]) have shown that there is no single MSA tool that consistently outperforms the rest in producing reliable phylogenetic trees. Moreover, Golubchik et al. ([3]) showed that the absence of amino acid residues often leads to an incorrect placement of gaps in the alignments, even when the sequences were otherwise identical, and, for a given alignment, not all amino acid positions will be aligned with equal confidence [4].

The identification of characteristic short amino acid sequences has been suggested as an efficient way of retrieving and classifying genes belonging to specific gene lineages (see for instance [5]). The presence of characteristic gap alignments has been also suggested as a diagnostic feature (see for instance [6]), but this approach relies on the assumption that the alignment is not ambiguous. Gene structure may also be used as a way to classify genes (see for instance [7]).

Synteny is often used to corroborate the inferred phylogeny or to suggest an alternative explanation for the data that is not supported by the inferred phylogeny. Although the genomes of many species are now available, the annotation process is far from being completed. An apparent lack of synteny can be due to an incomplete annotation of the genome. Since a large fraction of genes in any genome are still labeled as unknown or putative, the use of synteny is often far from trivial.

In this work, we assess the utility of using diagnostic amino acid residues in evolutionary studies using the *distal*-*less* (*DLX*) gene family as a test case. *DLX* genes belong to the large animal homeobox gene family, and play important roles in bilaterian and non-bilaterian embryonic development [8].

A single *DLX* gene is found in non-bilaterian animals [9] and in all Protostomes [10] studied to date. Three genes are found in the Urochordate *Ciona intestinalis*, with two of them arranged as a convergently transcribed bi-gene cluster [11]. In lampreys, four *DLX* genes are found but only one convergently transcribed bi-gene cluster has been identified [10], [12]. In vertebrates, three convergently transcribed bi-gene clusters are observed [10], [11]. In some vertebrate species, however, additional *DLX* copies can be found that are not arranged as bi-gene clusters [10], [11], [13], [14].

The above observations suggest that a *DLX* tandem gene duplication most likely occurred after the separation of the Cephalocordata and Urochordata/Vertebrata species. Furthermore, that the three *DLX* bi-gene clusters observed in vertebrate species are the result of two rounds of genome duplication, followed by the loss of one *DLX* bi-gene cluster [10]. The additional *DLX* copies that are not arranged as bi-gene clusters may be the result of single copy *DLX* duplications.

In order to corroborate the proposed evolutionary scenario for this gene family, Stock ([10]) performed phylogenetic analyses using a limited number of *DLX* gene sequences. Although the proposed evolutionary hypothesis predicts that all Urochordate *DLX* genes should be members of one or the other of the two main clades of vertebrate *DLX* genes (*DLX* genes *1*/*4*/*6* and *DLX 2*/*3*/*5*), only *DLXc* weakly clusters with the *DLX 1/4/6* clade, while *DLXa* and *DLXb* genes do not cluster with members of one of the two main clades. One out of the three Urochordate genes is predicted to be a duplication of one of the two genes in the Urochordate bi-gene cluster but the phylogenetic analyses reported by Stock do not support this view. Surprisingly, in Stock's phylogenetic analyses the Urochordate *DLXa* gene clusters with Protostome and amphioxus genes.

In this work we evaluate whether the use of a large number of gene sequences from a wide range of animal species, as well as the use of multiple alternative alignments, and the use of amino acids aligned with high confidence only, is enough to recover the expected *DLX* evolutionary history. Furthermore, on the basis of the current genome annotation, the use of synteny information for large scale studies is also considered. The identification of characteristic amino acid residues in the homeodomain region (a region where the alignment is generally not ambiguous; see for instance [6]) is also considered as an alternative/complementary gene identification approach.

## Materials and Methods

### Identification of informative homeodomain amino acid residues

In order to identify fixed (or almost fixed) amino acid differences between *DLX* genes, as well as between *DLX* clades *1*/*4*/*6* and *2*/*3*/*5*, the *DLX* data set compiled by ENSEMBL, containing 370 putative *DLX* sequences (ENSEMBL's database protein family: ENSF00000000699; ENSEMBL release 48) was used. Partial homeodomain *DLX* sequences were not used. Very divergent sequences that could not be unambiguously aligned with all other *DLX* sequences were also excluded. Non-annotated sequences that, at the amino acid level, and in the homeodomain region, were identical to annotated sequences were also used, and were given the same annotation as the annotated sequence(s). In few cases, sequences labeled as different *DLX* genes had identical homeodomain amino acid sequence. For instance, although the *Bos taurus* entry ENSBTAP00000044029 is labeled as *DLX1*, the corresponding homeodomain amino acid sequence is identical to the homeodomain sequence of 19 *DLX2* sequences from mammalian and amphibian species. These are very likely annotation mistakes and were treated as such. Table S1 shows ENSEMBL's accession numbers for the used *DLX* amino acid sequences.

*Synteny analyses*: In order to efficiently retrieve synteny information centered on a given *DLX* gene, a web-based application was developed (http://evolution.ibmc.up.pt/~nf/ensyntex/). This application accepts both ENSEMBL's gene, transcript or protein accession numbers. Only two parameters must be specified, namely the number of genes to be reported on either side of the reference gene (*N*) and the size of the region (in Kb) to be considered (*S*). For this study we used *N* = 2 and *S* = 500. A single annotated *DLX* protein accession was used per gene.

### Phylogenetic analyses

**Figure 1:**
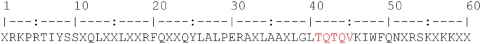
Canonical *DLX* sequence. The *DLX* specific TQTQV motif is highlighted in red.

The NCBI protein database, as well as ENSEMBL database (release 53), were queried using BLASTP and the canonical *DLX* sequence presented in Figure 1. The non-default settings used for BLASTP in NCBI and ENSEMBL were: W = 2, E = 0.001, and Max.Sequences (B) = 10000. Sequences that did not have a start and stop codon or the *DLX* characteristic motifs TQTQV/TQTQI/SQTQV (see Results) were discarded. The final dataset contains 222 different sequences. (sequence identifiers can be found in Table S2).

The multiple alignment algorithms implemented in the following software were used to align the amino acid sequences: M-Coffee [15], T-coffee [4], and Muscle [16]. Furthermore, as suggested by Notredame *et al.* ([4]), we considered only amino acid aligned positions without gaps and with a score greater than 3. The number of such positions obtained using M-coffee, T-coffee, and Muscle was, respectively, 43, 48, and 39. The amino acid alignments were used as a guide to obtain the corresponding nucleotide alignment and only the nucleotide positions corresponding to amino acid positions with a score greater than 3 were used in the phylogenetic analyses.

In order to infer the relationship of the 222 nucleotide sequences retrieved from NCBI and ENSEMBL, a fast maximum likelihood method of tree reconstruction, as implemented in GARLI [17], was used with the default options. The model used was the generalized time-reversible (GTR) model of sequence evolution, allowing for among-site rate variation and a proportion of invariable sites. For large datasets containing very divergent sequences, as it is here the case, this is almost always the best fit model of sequence evolution [17]. Majority-rule consensus trees were computed using five hundred trees that resulted from five hundred independent executions of GARLI.

## Results

### A simple method for the retrieval of DLX genes

In order to establish a simple method for the retrieval of *DLX* sequences only, the large set of *DLX* sequences compiled by ENSEMBL was used to derive a canonical sequence for the *DLX* homeodomain (Fig. 1). This sequence can be used to query public databases using BLAST. It should be noted that mammalian *DLX6* sequences can differ in as many as 9 positions from the canonical sequence. On the other hand, the *DLX* homeodomain sequence from *Nematostela vectensis* (a non-bilaterian cnidarian; accession ABB86447) differs from the canonical sequence at a single position.

When using a large homeodomain data set, Fonseca et al. [18] identified a region that can be used to classify HOX genes (homeodomain amino acid residues 41 to 45). In this region, the vast majority of *DLX* sequences compiled by ENSEMBL show the TQTQV amino acid motif, including one sequence from the non-bilaterian species *Trichoplax adhaerens*. The same is true for the *Nematostela vectensis DLX* homeodomain sequence (not included in the ENSEMBL dataset; accession ABB86447).

The exceptions are the *Dasypus novemcinctus DLX4*, *Takifugu rubripes DLX1*, *Oryctolagus cuniculus DLX6*, *Caenorhabditis* sp., and *Ciona* sp. *DLXa* gene sequences. It should be noted that, at this stage, it is not possible to rule out sequencing errors as the cause of the observed exceptions. Nevertheless, the *Caenorhabditis* sp., and *Ciona* sp. *DLXa* sequences are supported by multiple entries from different species. The TQTQV, TQTQI and SQTQV motifs (the latter two present in several sequences, and thus likely not sequencing errors) are absent from the sample of more than 1200 non-*DLX* homeodomain amino acid sequences analyzed by Fonseca et al. [18] from the HoxL, NKL, PRD, LIM, POU, HNF, SINE, TALE, CUT, PROS, ZF, and CERS classes (data not shown).

Given the observation that the TQTQV/TQTQI/SQTQV motifs do not occur outside the *DLX* gene family, it is very likely that these motifs are functionally important in *DLX* genes. Therefore, *DLX* genes can be easily identified in non-annotated genomes by looking for sequences showing homology with the canonical DLX homeodomain sequence, and then by filtering for those showing these motifs.

### Synteny as a tool for DLX gene identification in species containing multiple DLX genes

When using very divergent species and gene sequences, synteny is often used to corroborate the inferred phylogeny or even to suggest an alternative explanation for the data that is not supported by the inferred phylogeny. Table 1 shows the gene names that are most often associated with a given *DLX* gene. Genes without a proper name, i.e, those with a general identifier only, cannot be considered when doing large scale studies, since they likely have different identifiers in different species. Therefore, additional time consuming analyses would need to be performed in order to confirm the possible orthology of different gene sequences. In our study we use ENSEMBL's gene annotations to derive the synteny information. Note that ENSEMBL already provides synteny information at the chromosome level only, unfortunately this excludes many genomes.

**Table 1:** Annotated flanking genes (number of occurrences in brackets) in the close vicinity of *DLX* genes.

*DLX* gene	Annotated flanking genes
*DLX1* (24)	*DLX2* (14), *Hat1* (12), *Itga6* (8), *U6* (2), *Metapl1* (2), *Slc25a12* (2)
*DLX2* (13)	*DLX1* (10), *Itga6* (7), *Pdk1* (2)
Teleosteii *DLX2a* (2)	*DLX1a* (2)
*DLX3* (15)	*DLX4* (12), *Itga3* (7), *Pdk2* (6), *Myst2* (3), *Ace* (2), *Samd14* (2), *U6* (2)
*DLX4* (16)	*DLX3* (9), *Itga3* (7), *Tac4* (4), *Myst2* (3), *Slc35b1* (2), *U6* (2)
Teleosteii *DLX4a* (2)	
Teleosteii *DLX4b* (2)	*DLX3* (2), *Myst2* (2)
*DLX5* (22)	*DLX6* (14), *Acn9* (11), *Shfm1* (5), *Taq1* (2), *Eif2c2* (2), *Mpp6* (2)
*DLX6* (22)	*DLX5* (18), *Acn9* (11), *Shfm1* (9), *Eif2c2* (2), *Slc25a13* (2)

From the results presented in Table 1 it is clear that synteny can be useful as a tool for gene identification. However, the retrieved information greatly depends on genome annotation. It should be noted that the identification of *DLX* genes using synteny information depends on the correct identification of genes that also belong to large gene families such as *Itga* and *Slc* genes. The most important issue is that not all genomes are equally well annotated. In Urochordate species, in the region where *DLX* genes (*DLXa*, *DLXb* and *DLXc*) are located, genes have only a general identifier, thus Urochordate *DLX* genes are not shown in Table 1.

### A simple method for the identification of vertebrate DLX genes

The homeodomain region is in the vast majority of cases possible to align unambiguously ([6]). Therefore, it is of interest to determine whether, in this region, there are characteristic amino acid residues that allow an easy classification of vertebrate *DLX* genes. Since a *DLX* tandem gene duplication is likely involved in the origin of the *DLX* bi-gene cluster, we also looked at amino acid residues that may be characteristic of one or the other main *DLX* gene classes (*DLX 1*/*4*/*6* and *DLX 2*/*3*/*5*, (the vertebrate bi-gene clusters are: *DLX1*/*2*; *DLX 4*/*3*; *DLX 6*/*5*; [10]). The results are presented in Tables 2 and 3.

**Table 2:** Fixed amino acid differences between a given *DLX* gene and all other *DLX* genes (excluding fast evolving sequences).

Gene	Amino acid position				
	17	18	23	56	60
*DLX2*					W
*DLX3*			A		Y
*DLX4* a		Q^c^		Y^d^	
*DLX4b* b	H				
*DLX5*				I,L,M	
*DLX6*		H^e^			

a including Teleostei sequences.

b sequences from Teleostei.

c An H is observed in the Elasmobranchii *DLX4* sequence.

d Not observed in *DLX4 Ornithorhynchus anatinus*. Amphibian *DLX1* and *DLX6* sequences also show a Y at this position.

e An H is also observed in Amphibian *DLX1* sequences and in the Elasmobranchii *DLX4* sequence.

**Table 3:** Fixed amino acid differences between *DLX* genes *1*/*4*/*6* and *2*/*3*/*5*.

*DLX* genes	Amino acid position^a^		
	11	14	17
*DLX 1*/*4*/*6*	L, **V**	Q	N, H, **K**
*DLX 2*/*3*/*5*	F, Y	A	Q
Protostomes	L, **I**	Q	N

a In bold are shown rare (three or less occurrences) amino acid variants.

Table 2 shows that there are characteristic amino acid residues for *DLX2*, *DLX3*, *DLX4*, *DLX4b* (in Teleostei), *DLX5* and *DLX6*. Therefore, it is possible to classify *DLX* sequences in species with 3 bi-gene clusters (excluding Teleostei) by looking at four amino acid sites only (positions 18, 23, 56 and 60). *DLX1* sequences are classified as sequences that do not fit the rules for the other *DLX* genes. In Teleostei fish where eight *DLX* genes are found, it is possible to distinguish *DLX4a* from *DLX4b* by looking at position 17. It is not possible to distinguish *DLX2a* from *DLX2b* because at the amino acid level, in the homeodomain region, sequences from the two genes are always identical. The *Triakis semifasciata DLX* genes *1* to *6* follow the pattern described in Table 2, with the exception of the DLX4 amino acid sequence that does not show a Q at position 18.

Three differences are observed between the two main *DLX* gene classes (at sites 11, 14 and 17; Table 3). For these sites it is possible to infer the ancestral state by comparison with Protostome *DLX* sequences. *DLX* genes *1*/*4*/*6* show the ancestral amino acid variant at these positions.

In conclusion, the information given on Tables 1 and 2 can be combined to identify all *DLX* genes but the Teleostei genes *DLX2a* and *DLX2b* since the homeodomain amino acid sequence of these two genes is identical.

### Testing Stock's evolutionary hypothesis

Stock's hypothesis regarding the evolution of *DLX* genes was not strictly based on the interpretation of the phylogenetic analyses presented by this author. For instance, the placement of Urochordate sequences offered little or no support for the proposed evolutionary scenario (see Introduction). When using the informative amino acid positions reported in Table 3, Urochordate genes cannot be unambiguously classified into one of the two *DLX* gene clades. Discrepancies are, however, expected, since it is conceivable that informative changes may have occurred after the divergence of the Urochordates but before the appearance of the lamprey lineage.

**Figure 2:**
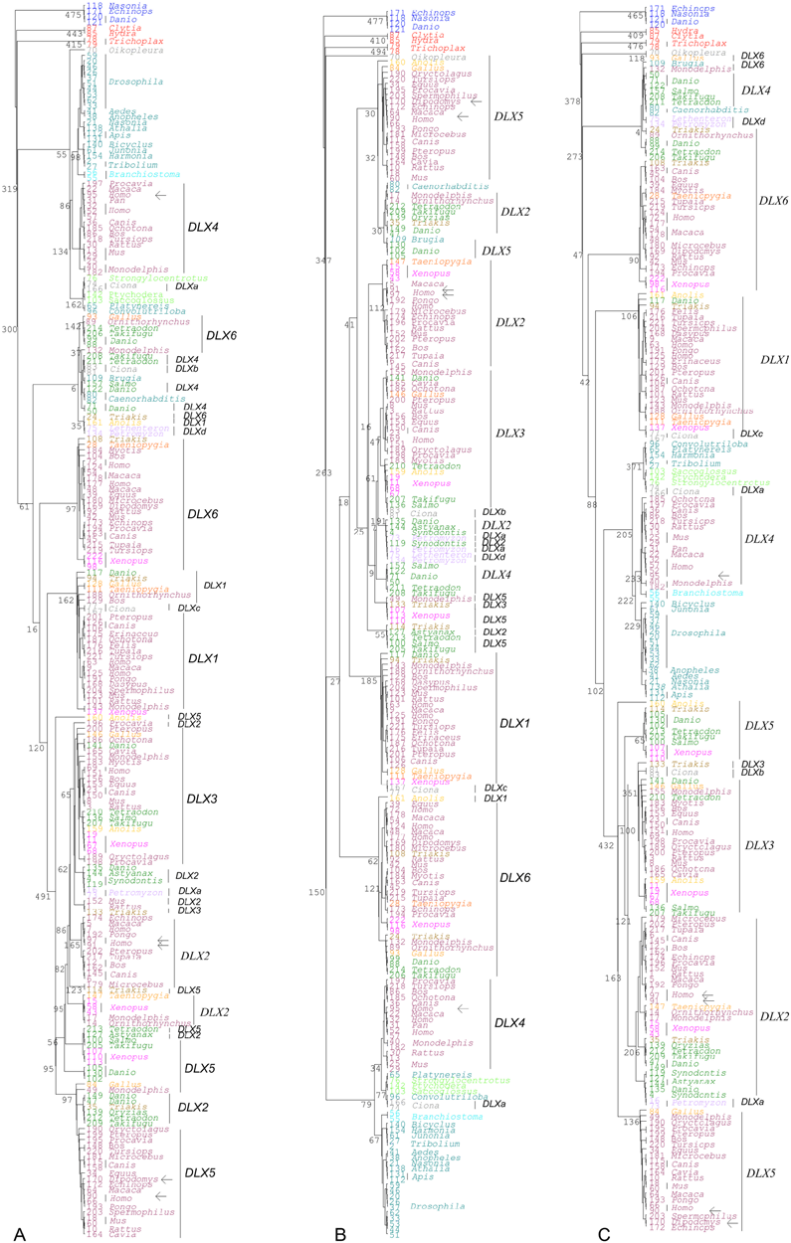
Maximum likelihood phylogenetic trees based on a set of 222 sequences (four *NK2* and 218 *DLX* sequences) aligned using different multiple alignment algorithms. A) M-Coffee [15]; B) Muscle [16]; C) T-coffee [4]. As suggested by Notredame *et al.* ([4]), only amino acid aligned positions without gaps and with a score greater than 3 were used. Numbers are the number of times a given cluster is obtained out of 500 replicates. Blue – *NK2* gene sequences; Red – Non-bilaterian species; Plum – Mammals; Light orange – Aves; Green – Teleostei fish; Gray 50% - Urochordata; Teal – Protostomes; Gold – Reptiles; Brown – Elasmobranchii; Light green – Hemichordata; Turquoise – Cephalocordata; Pink – Amphibians; Lavender – Hyperoartia. Arrows point to sequences that do not show the region where the characteristic *DLX* amino acids are located.

We next assess whether the use of a large number of gene sequences from a wide range of animal species, as well as the use of multiple alternative alignments, and the use of amino acids aligned with high confidence only, is enough to recover the expected *DLX*'s evolutionary history. Results are shown in Fig. 2. The sequences here used were obtained by BLAST, using the canonical *DLX* sequence described above, and by filtering for those that show the TQTQV/TQTQI/SQTQV motifs. A set of 222 non-redundant nucleotide sequences, containing a start and a stop codon, obey the above criteria. It should be noted that although this set is non-redundant, the same gene from the same species may be represented more than once in the phylogeny due to the presence of polymorphism, sequencing errors, or presence of alternative spliced forms. Four sequences present one of these motifs but not at the expected place. Therefore, the false positive error rate is 1.8% when information on where the motif occurs is not used. All four sequences are annotated as belonging to the *NK2* gene family. These four sequences were used to root the cladogram shown in Fig. 2. As expected, *DLX* sequences from non-bilaterians (phylum Cnidaria and Placozoa) are at the base of the *DLX* phylogeny. Protostome *DLX* sequences were expected to be the next most basal group. Although in two out of the three phylogenetic trees shown in Fig. 2, Protostome sequences tend to be basal, they are shown intermingled with Deuterostome sequences. Moreover, although the *DLX 2/3/5* clade is highly supported in two out of the three phylogenetic trees, there is little evidence for the presence of an *DLX 1/4/6* clade.

## Discussion

Large scale studies using hundreds of sequences can give a more detailed picture of the evolution of a given gene family. For instance, in principle, it can highlight duplication and amino acid substitution events that may correlate with the evolution of novel evolutionary features. Nevertheless, retrieving only the sequences of interest by BLAST from public databases can be a challenging problem when working with highly divergent species. Here we show that, at least for the *DLX* gene family, the characteristic homeodomain amino acid motifs (TQTQV/TQTQI/SQTQV) can be used to only retrieve the sequences of interest. It should be noted that the homeodomain region can be usually non-ambiguously aligned [6]. Further work must be conducted to determine whether such an approach works for any homeobox gene and for non-homeobox genes. However, it should be noted that Vieira *et al.* [5] presented characteristic amino acid motifs that allow the identification of plant *T2-RNases* belonging to a lineage that is at least 120 million years old. Those characteristic amino acid patterns were found in the plant *T2-RNase* conserved active site region.

The use of sequence information from non-annotated, non-curated, or partially annotated genomes poses also challenging problems, regarding gene identification. A phylogenetic approach can and should be used to classify gene sequences. Nevertheless, when using very divergent species many unexpected features are likely to be shown in the inferred phylogenetic trees, as here shown for the *DLX* family test case.

When genome annotation is available, synteny can be used to confirm gene classification. A web-based program for the easy retrieval of synteny information is here made available. Nevertheless, at present, it is still impractical to use information from genes that are identified by general identifiers only. As an alternative/complementary procedure, we have shown that DLX sequences can be accurately classified using characteristic amino acid residues, located in a region of the protein that can be usually non-ambiguously aligned (such as the homeobox region; [6]). Further work must be conducted to determine whether such an approach works for any homeobox containing gene and for non-homeobox genes.

The three *DLX* bi-gene clusters observed in higher vertebrates have been proposed to be the result of a tandem duplication followed by two rounds of genome duplication and the loss of one *DLX* bi-gene cluster [10]. Urochordata are the sister group of Vertebrata while Cephalocordata are the sister group to Urochordata/Vertebrata [19]. In Cephalocordata species there is no *DLX* bi-gene cluster while in Urochordata species there is a single *DLX* bi-gene cluster. Thus, the *DLX* tandem gene duplication most likely occurred after the separation of the Cephalocordata and Urochordata/Vertebrata species. All *DLX* genes from Urochordata and Vertebrata species must thus belong to one of the two gene lineages defined by the *DLX* tandem duplication [10]. It should be noted that, in the phylogeny shown in Fig. 2, there is little evidence for a *DLX 2/3/5* and a *DLX 1/4/6* clade, but there are fixed amino acid differences between genes from the two clades (see Table 3). In lampreys (Vertebrata), where a single bi-gene cluster is present, the *DLXd* gene sequences follow the pattern shown in Table 1 for *DLX 1*/*4*/*6*, while *DLXa*, *b*, and *c* show the pattern for *DLX 2*/*3*/*5* gene sequences. Thus, the differentiation between the two genes of the ancestral bi-gene cluster ended before the appearance of the lamprey lineage. Nevertheless, Urochordata *DLX* genes cannot be unambiguously classified using the information given on Table 3. *DLXc* shows the ancestral state at all positions listed in Table 3. It could be thus, classified as belonging to the *DLX 1*/*4*/*6* clade. This interpretation depends, however, on the assumption that the differentiation between the two *DLX* genes of the ancestral bi-gene cluster started before the appearance of the Urochordata lineage.

In the phylogenetic analyses here presented *DLXc* is shown as being closely related to *DLX1* sequences thus offering some support to the hypothesis that it does belong to the *DLX 1*/*4*/*6* clade. *DLXa* shows the derived amino acid residue at position 11. It could be thus tentatively classified as belonging to the *DLX 2*/*3*/*5* lineage, although it does not present the derived amino acid residue at position 14. At position 17 it uses an amino acid residue (V) not used in Vertebrate *DLX* genes. Nevertheless, in the phylogenetic analyses *DLXa* always clusters with Protostome *DLX* sequences and Deuterostome *DLX4* sequences. *DLXb* is difficult to classify since it shows putatively derived amino acid residues at position 11 and 17. The first one suggests that this gene belongs to the *DLX 2*/*3*/*5* lineage while the second one suggests that it belongs to the *DLX 1*/*4*/*6* lineage. It could be that the same amino acid substitution appeared twice during evolution. Nevertheless, a non-homologous recombination event that creates a chimeric gene cannot be ruled out either. Depending on the alignment, *DLXb* clusters with sequences of the *1*/*4*/*6* or *2*/*3*/*5* clade. In any case, the extreme conservation of the amino acid differences found between *DLX* genes *1*/*4*/*6* and *2*/*3*/*5* suggests that these changes are functionally important. It should be noted that there are no fixed amino acid differences between Protostome and Deuterostome *DLX* sequences because a fraction of the genes that belong to the *DLX 1*/*4*/*6* lineage (but not those that belong to the *DLX 2*/*3*/*5* lineage) show the ancestral state at positions 11, 14 and 17 (Table 3). None of these positions are part of the recognition helix 3 that is essential for successful and specific DNA binding [20]. The identification of gene and gene lineage characteristic amino acids will also help focus experimental studies onto investigating the biochemical functions of key *DLX* amino acid residues.

## Supporting Information

Table S1 DLX accession numbers.
(0.06 MB DOC)

Table S2 Accession numbers for the sequences used in the phylogenetic analyses (see Figure 2).
(0.16 MB DOC)
