# Supplementary material for: Gene Classification Based on Amino Acid Motifs and Residues: The DLX (distal-less) Test Case
Source: PLoS One. 2009 Jun 1;4(6):e5748. doi: 10.1371/journal.pone.0005748 (PMC2685005; doi:10.1371/journal.pone.0005748)
Supplement: Table S1 — DLX accession numbers. (0.06 MB DOC) [file pone.0005748.s001.doc]

| Gene^a^ | Species and accession number |
| --- | --- |
| *DLX1* | *Bos taurus* (A6H733); *Bos taurus* (ENSBTAP00000020246); *Mus musculus* (ENSMUSP00000042413); *Rattus norvegicus* (ENSRNOP00000002078); *Tetraodon nigroviridis* (GSTENP00031429001); *Tetraodon nigroviridis* (Q4RNS8); *Canis lupus familiaris* (ENSCAFP00000019056); *Dasypus novemcinctus* (ENSDNOP00000009607); *Erinaceus europaeus* (ENSEEUP00000002304); *Felis catus* (ENSFCAP00000002020); *Gasterosteus aculeatus* (ENSGACP00000006516); *Gallus gallus* (Q6DV98); *Homo sapiens* (ENSP00000354478); *Homo sapiens* (ENSP00000354865); *Homo sapiens* (ENSP00000376360); *Homo sapiens* (P56177); *Homo sapiens* (Q53SU3); *Loxodonta africana* (ENSLAFP00000002959); *Monodelphis domestica* (ENSMODP00000011081); *Macaca mulatta* (ENSMMUP00000009589); *Microcebus murinus* (ENSMICP00000006353); *Mus musculus* (A2ATU7); *Mus musculus* (Q64317); *Ornithorhynchus anatinus* (ENSOANP00000013669); *Ornithorhynchus anatinus* (ENSOANP00000013670); *Otolemur garnettii* (ENSOGAP00000010643); *Oryzias latipes* (ENSORLP00000021716); *Ochotona princeps* (ENSOPRP00000014210); *Pan troglodytes* (ENSPTRP00000039172); *Spermophilus tridecemlineatus* (ENSSTOP00000011616); *Tupaia belangeri* (ENSTBEP00000008284); *Triakis semifasciata* (Q5MLG5); *Xenopus tropicalis* (A0PJP5); *Xenopus tropicalis* (ENSXETP00000016415); *Danio rerio* (ENSDARP00000011033); *Danio rerio* (Q98875); *Sorex araneus* (ENSSARP00000011165); *Takifugu rubripes* (SINFRUP00000149398); *Xenopus laevis* (Q3KQH4); *Xenopus laevis* (Q4QR07); *Xenopus laevis* (P53773); *Ornithorhynchus anatinus* (ENSOANP00000002711); |
| *DLX2* | *Xenopus laevis* (Q06403); *Triakis semifasciata* (Q5MLG4); *Bos taurus* (ENSBTAP00000007545); ***Bos taurus* (*DLX1* - ENSBTAP00000044029)**; *Canis lupus familiaris* (ENSCAFP00000031627); *Erinaceus europaeus* (ENSEEUP00000013906); *Echinops telfairi* (ENSETEP00000014271); *Homo sapiens* (ENSP00000234198); *Homo sapiens* (Q07687); *Homo sapiens* (Q53QU7); *Macaca mulatta* (ENSMMUP00000023156); *Microcebus murinus* (ENSMICP00000006356); *Mus musculus* (ENSMUSP00000024159); *Mus musculus* (P40764); *Mus musculus* (Q52KJ2); *Ornithorhynchus anatinus* (ENSOANP00000013671); *Ornithorhynchus anatinus* (ENSOANP00000013673); *Rattus norvegicus* (ENSRNOP00000002076); *Tupaia belangeri* (ENSTBEP00000008308); *Xenopus tropicalis* (ENSXETP00000016414); *Xenopus tropicalis* (Q66JE3); *Monodelphis domestica* (ENSMODP00000011090); *Takifugu rubripes* (SINFRUP00000149399); *Astyanax mexicanus* (Q0PJT7); *Danio rerio* (ENSDARP00000020230); *Danio rerio* (Q503J7); *Synodontis multipunctatus* (Q0PJT0); *Astyanax mexicanus* (Q0PJT6); *Danio rerio* (A4IG28); *Danio rerio* (ENSDARP00000007080); *Danio rerio* (Q98876); *Synodontis multipunctatus*(Q0PJS9); *Danio rerio* (P50574); *Gasterosteus aculeatus* (ENSGACP00000006506); *Oryzias latipes* (ENSORLP00000021729); *Oryzias latipes* (ENSORLP00000021730); *Oryzias latipes* (Q0PJT1); |
| *DLX3* | *Astyanax mexicanus* (Q66PH5); ***Xenopus laevis* (*DLX2* - P53774)**; *Ambystoma mexicanum* (Q90229); *Gasterosteus aculeatus* (ENSGACP00000013056); *Gallus gallus* (Q9PT89); *Notophthalmus viridescens*(P53770); *Oryzias latipes* (ENSORLP00000005152); *Pleurodeles waltlii* (Q91284); *Xenopus tropicalis* (Q28BP8); *Xenopus tropicalis* (Q5BL47); *Danio rerio* (ENSDARP00000022291); *Danio rerio* (Q01702); *Danio rerio* (Q6DBS2); *Takifugu rubripes* (SINFRUP00000127097); *Bos taurus* (A2VDN3); *Bos taurus* (ENSBTAP00000023142); *Canis lupus familiaris* (ENSCAFP00000019066); *Canis lupus familiaris* (ENSCAFP00000024978); *Eleutherodactylus coqui* (Q2KT32); *Homo sapiens* (ENSP00000172196); *Homo sapiens* (O60479); *Myotis lucifugus* (ENSMLUP00000006648); *Macaca mulatta* (ENSMMUP00000000217); *Microcebus murinus* (ENSMICP00000015055); *Mus musculus* (ENSMUSP00000090443); *Mus musculus* (Q64205); *Mus musculus* (Q78ZZ8); *Mus musculus* (Q9QWH3); *Oryctolagus cuniculus* (ENSOCUP00000006768); *Ochotona princeps* (ENSOPRP00000011933); *Pan troglodytes* (ENSPTRP00000015940); *Pan troglodytes* (ENSPTRP00000054336); *Rattus norvegicus* (ENSRNOP00000005797); *Monodelphis domestica* (ENSMODP00000015153); *Takifugu rubripes* (SINFRUP00000139570); *Triakis semifasciata* (Q5MLG3); |
| *DLX4* | *Xenopus tropicalis* (ENSXETP00000047431); *Monodelphis domestica* (ENSMODP00000015160); *Myotis lucifugus* (ENSMLUP00000006641); *Bos taurus* (ENSBTAP00000009672); *Microcebus murinus* (ENSMICP00000015051); *Rattus norvegicus* (ENSRNOP00000005807); *Canis lupus familiaris* (ENSCAFP00000024973); *Echinops telfairi* (ENSETEP00000007288); *Gorilla gorilla* (A1YEZ9); *Homo sapiens* (A6NML4); *Homo sapiens* (ENSP00000240306); *Homo sapiens* (Q92988); *Loxodonta africana* (ENSLAFP00000011265); *Macaca mulatta* (ENSMMUP00000023235); *Mus musculus* (ENSMUSP00000021241); *Mus musculus* (P70436); *Macaca nemestrina* (A2T6N5); *Otolemur garnettii* (ENSOGAP00000004594); *Pan paniscus* (A1YG68); *Pan troglodytes* (A2T745); *Pan troglodytes* (ENSPTRP00000015938); *Pan troglodytes* (ENSPTRP00000015939); *Tupaia belangeri* (ENSTBEP00000013450); *Dasypus novemcinctus* (ENSDNOP00000002868); *Macaca mulatta* (A2D688); *Triakis semifasciata* (Q5MLG2); *Xenopus laevis* (P53775); *Danio rerio* (ENSDARP00000096665); *Danio rerio* (Q98878); *Oryzias latipes* (ENSORLP00000005139); *Takifugu rubripes* (SINFRUP00000127095); *Gasterosteus aculeatus* (ENSGACP00000006762); *Tetraodon nigroviridis* (GSTENP00021076001); *Tetraodon nigroviridis* (Q4SB92); *Danio rerio* (ENSDARP00000021032); *Danio rerio* (Q98879); *Takifugu rubripes* (SINFRUP00000144960); *Danio rerio* (Q6DBX9); |
| *DLX5* | *Danio rerio* (ENSDARP00000062025); *Danio rerio* (P50576); *Danio rerio* (Q5XJL9); *Gasterosteus aculeatus* (ENSGACP00000008997); *Takifugu rubripes* (SINFRUP00000182572); *Oryzias latipes* (ENSORLP00000005733); *Triakis semifasciata* (Q5MLG1); *Microcebus murinus* (ENSMICP00000013170); ***Xenopus laevis* (*DLX3* - P54655)**; *Xenopus laevis* (Q6GLJ3); *Xenopus tropicalis* (ENSXETP00000036133); *Xenopus tropicalis* (Q6GLH9); *Bos taurus* (ENSBTAP00000024811); *Bos taurus* (Q1RMR7); *Canis lupus familiaris* (ENSCAFP00000003194); *Dasypus novemcinctus* (ENSDNOP00000006789); *Echinops telfairi* (ENSETEP00000010463); *Felis catus* (ENSFCAP00000010336); *Gallus gallus* (ENSGALP00000014846); *Gallus gallus* (ENSGALP00000015233); *Gallus gallus* (P50577); *Homo sapiens* (ENSP00000222598); *Homo sapiens* (P56178); *Homo sapiens* (Q53Y73); *Monodelphis domestica* (ENSMODP00000020564); *Monodelphis domestica* (ENSMODP00000027271); *Macaca mulatta* (ENSMMUP00000019575); *Mus musculus* (ENSMUSP00000052559); *Mus musculus* (P70396); *Mus musculus* (Q3TYA7); *Oryctolagus cuniculus* (ENSOCUP00000007432); *Oryctolagus cuniculus* (Q95JB0); *Pan troglodytes* (ENSPTRP00000033230); *Rattus norvegicus* (ENSRNOP00000014946); *Rattus norvegicus* (P50575); *Rattus norvegicus* (Q91WY7); *Spermophilus tridecemlineatus*(ENSSTOP00000004620); *Tupaia belangeri* (ENSTBEP00000005233); ***Ornithorhynchus anatinus* (*DLX6* - ENSOANP00000028201)**; *Erinaceus europaeus* (ENSEEUP00000010060); |
| *DLX6* | *Triakis semifasciata* (Q5MLG0); *Xenopus laevis* (Q0P3Q7); *Xenopus laevis* (Q52L43); *Xenopus laevis* (Q6DKA7); *Xenopus tropicalis* (ENSXETP00000036135); *Oryctolagus cuniculus* (ENSOCUP00000007444); *Bos taurus* (ENSBTAP00000028605); *Rattus norvegicus* (ENSRNOP00000014468); *Tetraodon nigroviridis* (GSTENP00032958001); *Tetraodon nigroviridis* (Q4RKH1); *Canis lupus familiaris* (ENSCAFP00000003190); *Cavia porcellus* (ENSCPOP00000001624); *Echinops telfairi* (ENSETEP00000010465); *Felis catus* (ENSFCAP00000010335); *Gasterosteus aculeatus* (ENSGACP00000008989); *Gallus gallus* (ENSGALP00000037211); *Gallus gallus* (ENSGALP00000037212); *Gallus gallus* (Q6DV99); *Homo sapiens* (ENSP00000007660); *Homo sapiens* (P56179); *Loxodonta africana* (ENSLAFP00000015103); *Monodelphis domestica* (ENSMODP00000020565); *Myotis lucifugus* (ENSMLUP00000015410); *Macaca mulatta* (ENSMMUP00000019574); *Microcebus murinus* (ENSMICP00000013153); *Mus musculus* (A5HKN2); *Mus musculus* (ENSMUSP00000031768); *Mus musculus* (P70397); *Oryzias latipes* (ENSORLP00000005708); *Pan troglodytes* (ENSPTRP00000033229); *Sorex araneus* (ENSSARP00000011601); *Tupaia belangeri* (ENSTBEP00000004813); *Takifugu rubripes* (SINFRUP00000178279); *Danio rerio* (ENSDARP00000062022); *Danio rerio* (ENSDARP00000093553); *Danio rerio* (Q29RC9); *Danio rerio* (Q98877); |
| *DLXa* | *Petromyzon marinus* (Q9DDF9); |
| *DLXb* | *Petromyzon marinus* (Q9DDF8); |
| *DLXc* | *Petromyzon marinus* (Q9DDF7); |
| *DLXd* | *Petromyzon marinus* (Q9DDF6); |
| *DLXa* | *Branchiostoma floridae* (P53772); |
| *DLXa* | *Ciona intestinalis* (Q9GP89); *Oikopleura dioica* (Q5QFC5); |
| *DLXb* | *Oikopleura dioica* (Q5EVL4); *Ciona intestinalis* (ENSCINP00000009044); *Ciona intestinalis* (ENSCINP00000022692); *Ciona intestinalis* (Q4H3Q0); *Ciona intestinalis* (Q9GP88); |
| *DLXc* | *Ciona savignyi* (ENSCSAVP00000004951); *Ciona intestinalis* (Q4H3P9); *Ciona intestinalis* (Q9GP87); *Ciona intestinalis* (ENSCINP00000014584); *Oikopleura dioica* (Q5GIY3); *Oikopleura dioica* (Q6E7C7); *Oikopleura dioica* (Q5EVL3); |
| *Protostome DLX* | *Platynereis dumerilii*(Q2WBX8); *Aedes aegypti* (AAEL001780PA); *Anopheles gambiae* (AGAP007058PA); *Drosophila melanogaster* (CG3629PA); *Drosophila melanogaster* (CG3629PB); *Aedes aegypti* (Q17K67); *Anopheles gambiae* (Q7PTF4); *Bicyclus anynana* (Q8WRT8); *Drosophila melanogaster* (A2VEF7); *Drosophila melanogaster* (P20009); *Drosophila pseudoobscura* (Q28Z29); *Harmonia axyridis* (Q2MHJ4); *Junonia coenia* (Q95VX3); *Junonia coenia* (Q9TX42); *Manduca sexta* (Q6IWM7); *Tribolium castaneum* (Q9GPL3); *Caenorhabditis elegans* (C28A5.4); *Caenorhabditis elegans* (Q18273); *Caenorhabditis elegans* (Q95QC6); *Caenorhabditis briggsae* (Q60YW3); *Tetranychus urticae* (A3QS76); *Ptychodera flava* (Q9U5E6); *Saccoglossus kowalevskii* (Q7YTB4); *Cupiennius salei* (Q9BI27); |
| Non-bilaterian *DLX* | *Trichoplax adhaerens* (Q27W44); *Trichoplax adhaerens* (Q27W45); |

^a^ ENSEMBL´s annotation is used. Sequences not annotated by ENSEMBL but that are identical in the homeodomain region to annotated sequences were also used (underlined). Sequences that in the homeodomain region are identical to other sequences but that have been identified as a different gene are shown in bold. These are likely classification errors.
