# Supplementary material for: Gene Classification Based on Amino Acid Motifs and Residues: The DLX (distal-less) Test Case
Source: PLoS One. 2009 Jun 1;4(6):e5748. doi: 10.1371/journal.pone.0005748 (PMC2685005; doi:10.1371/journal.pone.0005748)
Supplement: Table S2 — Accession numbers for the sequences used in the phylogenetic analyses (see Figure 2). (0.16 MB DOC) [file pone.0005748.s002.doc]

| Code | Accession number |
| --- | --- |
| 1 | XM_230986 |
| 2 | AF317551 |
| 3 | NM_001105832 |
| 4 | DQ822517 |
| 5 | XM_001086012 |
| 6 | XM_855787 |
| 7 | NM_004405 |
| 8 | NM_010055 |
| 9 | AB173282 |
| 10 | NM_012943 |
| 11 | NM_001097046 |
| 12 | XM_606914 |
| 13 | U73329 |
| 14 | XM_001514592 |
| 15 | NM_001008060 |
| 16 | AY010116 |
| 17 | XM_001368009 |
| 18 | NM_010056 |
| 19 | BC116461 |
| 20 | XM_002004592 |
| 21 | XM_001601822 |
| 22 | XM_001095208 |
| 23 | XM_860168 |
| 24 | AY738121 |
| 25 | NM_007867 |
| 26 | XM_002062924 |
| 27 | NM_001039439 |
| 28 | XM_002197324 |
| 29 | AK145123 |
| 30 | NM_001107040 |
| 31 | XM_511870 |
| 32 | NM_138281 |
| 33 | NM_001144287 |
| 34 | XM_001494149 |
| 35 | AY738119 |
| 36 | XM_846428 |
| 37 | XM_001986704 |
| 38 | XM_308706 |
| 39 | XM_001494576 |
| 40 | XM_001374214 |
| 41 | XM_001653942 |
| 42 | NM_010057 |
| 43 | NM_001095731 |
| 44 | S47947 |
| 45 | XM_539429 |
| 46 | XM_002048839 |
| 47 | NM_131311 |
| 48 | XM_001090683 |
| 49 | XM_001363044 |
| 50 | NM_131300 |
| 51 | XM_002092865 |
| 52 | U73328 |
| 53 | NM_079133 |
| 54 | XM_001090798 |
| 55 | XM_002212322 |
| 56 | XM_002212327 |
| 57 | NM_001934 |
| 58 | NM_001090563 |
| 59 | XM_002018373 |
| 60 | AF022075 |
| 61 | AF404110 |
| 62 | NM_166689 |
| 63 | BC036189 |
| 64 | XM_001090332 |
| 65 | AM114774 |
| 66 | NM_005221 |
| 67 | NM_001030395 |
| 68 | CR942714 |
| 69 | AK313257 |
| 70 | AY521597 |
| 71 | BC078319 |
| 72 | NM_005222 |
| 73 | AY010118 |
| 74 | NM_001032648 |
| 75 | AB048759 |
| 76 | NM_001129810 |
| 77 | NM_001032649 |
| 78 | XM_002117927 |
| 79 | DQ355802 |
| 80 | XM_001665654 |
| 81 | AB210372 |
| 82 | NM_065503 |
| 83 | NM_001032500 |
| 84 | NM_204159 |
| 85 | XM_002160199 |
| 86 | NM_001098041 |
| 87 | FJ392843 |
| 88 | BC114264 |
| 89 | XM_001521603 |
| 90 | AK297614 |
| 91 | AK316129 |
| 92 | XM_575377 |
| 93 | NM_001080890 |
| 94 | AY738118 |
| 95 | BC005812 |
| 96 | EU914949 |
| 97 | AK297503 |
| 98 | NM_001091836 |
| 99 | NM_131323 |
| 100 | NM_001140670 |
| 101 | BC167073 |
| 102 | BC083280 |
| 103 | AY318740 |
| 104 | XM_001254126 |
| 105 | BC165702 |
| 106 | XM_855727 |
| 107 | NM_001004778 |
| 108 | AY738123 |
| 109 | XM_001902346 |
| 110 | NM_001090564 |
| 111 | XM_002198751 |
| 112 | AB365073 |
| 113 | L09729 |
| 114 | AY738122 |
| 115 | XM_845319 |
| 116 | NM_001096815 |
| 117 | NM_131305 |
| 118 | XM_001600809 |
| 119 | DQ822516 |
| 120 | NM_131419 |
| 121 | BC079493 |
| 122 | NM_131318 |
| 123 | NM_010053 |
| 124 | AK094086 |
| 125 | NM_178120 |
| 126 | XM_855698 |
| 127 | DQ822509 |
| 128 | NM_001045842 |
| 129 | NM_001099017 |
| 130 | NM_131306 |
| 131 | NM_001131037 |
| 132 | XM_001363130 |
| 133 | AY738120 |
| 134 | AY010119 |
| 135 | NM_131297 |
| 136 | NM_001140828 |
| 137 | NM_001100257 |
| 138 | AB378321 |
| 139 | NM_001104820 |
| 140 | AF404825 |
| 141 | NM_131322 |
| 142 | AB028221 |
| 143 | XM_001367974 |
| 144 | DQ822510 |
| 145 | XM_845102 |
| 146 | NM_204804 |
| 147 | XM_002196034 |
| 148 | NM_001075313 |
| 149 | BC095303 |
| 150 | XM_548193 |
| 151 | NM_005220 |
| 152 | NM_010054 |
| 153 | XM_001918194 |
| 154 | AB200969 |
| 155 | XM_001367658 |
| 156 | NM_001081622 |
| 157 | NM_001140834 |
| 158 | XM_539430 |
| 159 | ENSACAP00000005005 |
| 160 | ENSACAP00000014450 |
| 161 | ENSACAP00000015892 |
| 162 | ENSBTAP00000044029 |
| 163 | ENSCAFP00000003190 |
| 164 | ENSCPOP00000001625 |
| 165 | ENSCPOP00000017373 |
| 166 | ENSCINP00000009039 |
| 167 | ENSCINP00000014582 |
| 168 | ENSDNOP00000009607 |
| 169 | ENSDORP00000009002 |
| 170 | ENSDORP00000009004 |
| 171 | ENSETEP00000000776 |
| 172 | ENSETEP00000010463 |
| 173 | ENSETEP00000010465 |
| 174 | ENSETEP00000014271 |
| 175 | ENSEEUP00000002304 |
| 176 | ENSFCAP00000002020 |
| 177 | ENSP00000007660 |
| 178 | ENSMMUP00000019574 |
| 179 | ENSMICP00000006356 |
| 180 | ENSMICP00000013153 |
| 181 | ENSMICP00000013170 |
| 182 | ENSMODP00000015160 |
| 183 | ENSMLUP00000006648 |
| 184 | ENSMLUP00000015410 |
| 185 | ENSOPRP00000011930 |
| 186 | ENSOPRP00000011933 |
| 187 | ENSOPRP00000014210 |
| 188 | ENSOANP00000013669 |
| 189 | ENSOCUP00000006768 |
| 190 | ENSOCUP00000007432 |
| 191 | ENSPPYP00000014438 |
| 192 | ENSPPYP00000014439 |
| 193 | ENSPPYP00000019945 |
| 194 | ENSPCAP00000006913 |
| 195 | ENSPCAP00000006954 |
| 196 | ENSPCAP00000008298 |
| 197 | ENSPCAP00000011847 |
| 198 | ENSPCAP00000011897 |
| 199 | ENSPVAP00000004229 |
| 200 | ENSPVAP00000006473 |
| 201 | ENSPVAP00000010683 |
| 202 | ENSPVAP00000010684 |
| 203 | ENSSTOP00000004620 |
| 204 | ENSSTOP00000011616 |
| 205 | ENSTRUP00000000590 |
| 206 | ENSTRUP00000018749 |
| 207 | ENSTRUP00000042330 |
| 208 | ENSTRUP00000042333 |
| 209 | ENSTRUP00000045465 |
| 210 | ENSTNIP00000014100 |
| 211 | ENSTNIP00000015832 |
| 212 | ENSTNIP00000020058 |
| 213 | ENSTNIP00000020912 |
| 214 | ENSTNIP00000020913 |
| 215 | ENSTBEP00000004813 |
| 216 | ENSTBEP00000008284 |
| 217 | ENSTBEP00000008308 |
| 218 | ENSTTRP00000002845 |
| 219 | ENSTTRP00000007183 |
| 220 | ENSTTRP00000007188 |
| 221 | ENSTTRP00000010783 |
| 222 | ENSXETP00000036135 |
